# Place cells in the claustrum remap under NMDA receptor control

**DOI:** 10.1111/ejn.15726

**Published:** 2022-06-17

**Authors:** Emanuela Rizzello, Seán K. Martin, Jennifer Rouine, Charlotte Callaghan, Mathias L. Mathiasen, Shane M. O'Mara

**Affiliations:** ^1^ School of Psychology and Institute of Neuroscience, Trinity College Dublin The University of Dublin Dublin Ireland; ^2^ School of Psychology Cardiff University Cardiff Wales UK; ^3^ Present address: Department of Veterinary and Animal Sciences University of Copenhagen Frederiksberg Denmark

## Abstract

Place cells are cells that exhibit location‐dependent responses; they have mostly been studied in the hippocampus. Place cells have also been reported in the rat claustrum, an underexplored paracortical region with extensive corto‐cortical connectivity. It has been hypothesised that claustral neuronal responses are anchored to cortical visual inputs. We show rat claustral place cells remap when visual inputs are eliminated from the environment, and that this remapping is NMDA‐receptor‐dependent. Eliminating visual input decreases claustral delta‐band oscillatory activity, increases theta‐band oscillatory activity, and increases simultaneously recorded visual cortical activity. We conclude that, like the hippocampus, claustral place field remapping might be mediated by NMDA receptor activity, and is modulated by visual cortical inputs.

## Graphical abstract

Claustral place cells were recorded during light manipulation while rats navigated in a square arena; cell positions were estimated by histological analyses. Claustral place cells exhibit light‐dependent location‐specific responses. This remapping is NMDA‐receptor‐dependent.

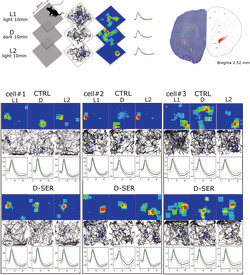

**Abbreviations**
COM: centre of mass
D‐SER: D‐serine
ISI: inter‐spike interval
LFP: local field potential
V2L: lateral secondary visual area cortex

## 1 INTRODUCTION

In mammals, several brain regions (including the hippocampus, anterior thalamus, and claustrum) contain cells whose firing is localized to ‘place fields’, which code the animal's position in an environment (see Grieves & Jeffery, 2017; OMara & Aggleton, 2019 for review). Place cells have been mostly investigated in the hippocampus, but there are reports of extra‐hippocampal place cells (Grieves & Jeffery, 2017; OMara & Aggleton, 2019). Jankowski and OMara (2015) reported spatially coding neurons responding to position, boundaries, object location, and head direction, across light and dark conditions in the claustrum; moreover, these spatially responsive claustral cells also responded to the rotation of visual distal cues in the environment.

Compared with the hippocampus, the claustrum is an under‐investigated paracortical region; it is adjacent to the orbitofrontal cortex (anteriorly), insular cortex (laterally), and the caudate nucleus of the striatum (medially). The claustrum is a thin sheet of neurons, possessing a complex dorsoventral and rostrocaudal topography, largely spanning the rostral half of the telencephalon (Dillingham et al., 2017). There are extensive connections between the claustrum and visual cortex (Pearson et al., 1982), including dense ipsilateral inputs from the secondary visual cortex (Miller & Vogt, 1984). We therefore reasoned claustral place field remapping might be affected by visual inputs. We manipulated visual inputs during freely moving navigation by foraging rats to explore potential mechanisms underpinning claustral place fields. We also measured oscillatory activity in the claustrum and visual cortex during light/dark manipulation. Place fields remained present but rapidly remapped by about 50° when visual inputs were eliminated by turning off the room lights. Previous experiments have shown place field remapping in the hippocampus is under NMDA receptor control (Kentros et al., 1998). We therefore modulated NMDA receptor activity via systemic D‐serine administration (an NMDA‐receptor agonist that readily passes the blood–brain barrier). First, we determine the D‐SER influences on local claustral network (LFP), finding an increase in total power following the drug administration. Second, we found the expected 50° remapping of claustral place cells when ambient visual inputs are eliminated was absent when D‐serine was administered. We also found exposure to darkness altered claustral oscillations in the theta and delta‐bands, while increasing visual cortical activity. We conclude that, like the hippocampus, claustral place field remapping might be mediated by NMDA receptor activity, and is modulated by visual cortical inputs.

## 2 RESULTS

After post‐mortem histological verification, 49 well‐isolated units recorded in 13 rats were assigned to the claustrum. Based on their electrophysiological and spatial properties, 40 units were classified as place cells (81.63%), and 9 as bursting cells without spatially related firing (18.37%; Table 1).

**TABLE 1:** Electrophysiological classification of claustral units (mean ± SEM)

	Place cells	Bursting cells
Width (μs)	249.20 ± 9.01	253.00 ± 84.33
Amplitude (μV)	134.89 ± 19.76	126.03 ± 42.01
ISI (ms)	3869.03 ± 968.24	1514.05 ± 618.10
Spike freq. (Hz)	0.35 ± 0.09	3.06 ± 1.02
Spatial Skaggs	2.54 ± 0.29	1.48 ± 0.49

*Note*: Summary statistics for mean spike width, mean spike amplitude, mean inter‐spike interval (ISI), mean spike frequency, and spatial information content (Skaggs) (mean ± SEM) for all claustral place and bursting cells recorded.

**FIGURE 1:**
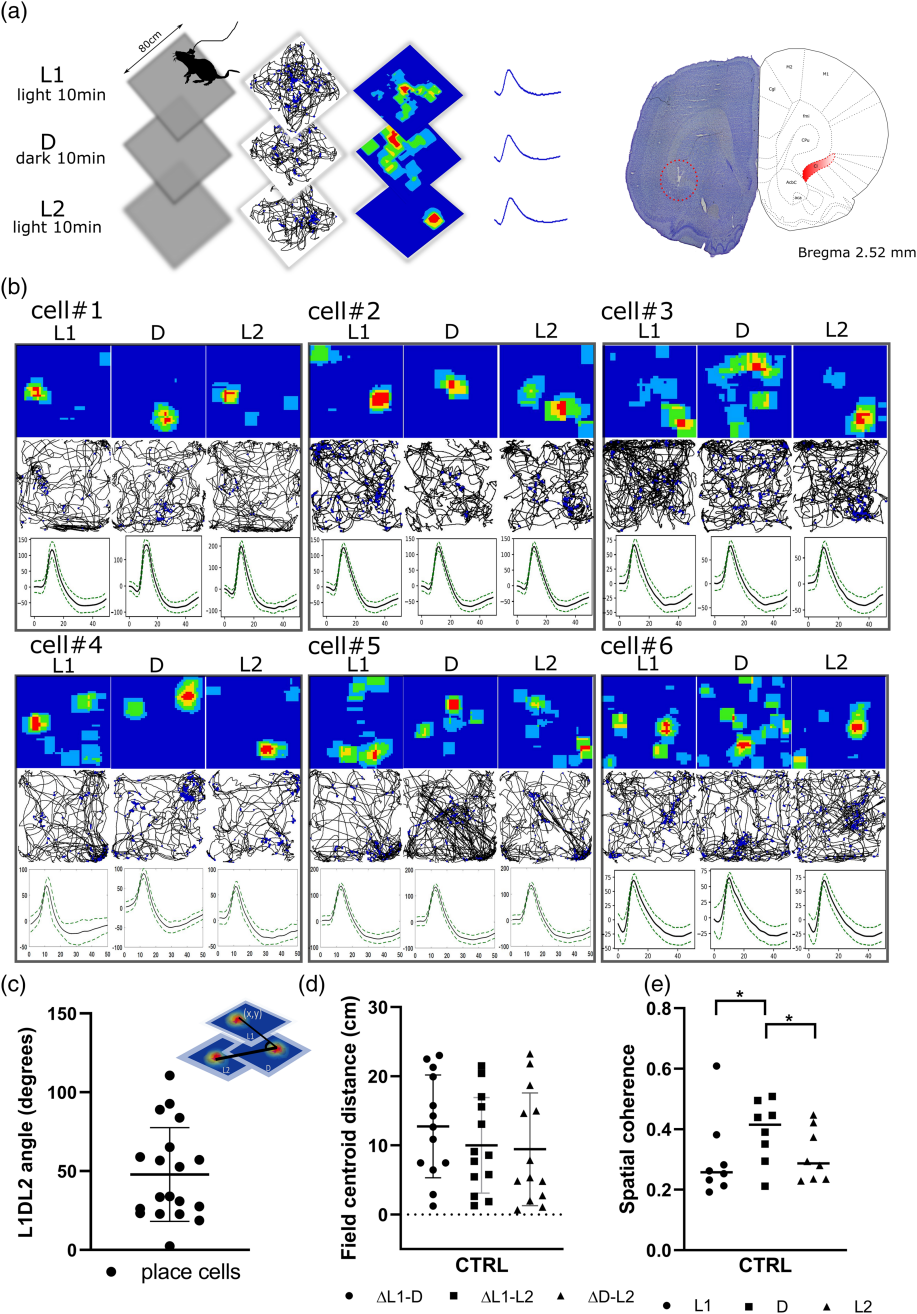
Claustral place cells remap in darkness. (a) On Day 1, claustral place cells were recorded over consecutive sessions (10 min each), during which animals navigated in the square arena (80 × 80 cm) with light manipulation (light [L1]/dark [D]/light [L2]); the figure shows an example of the animal's path recorded during navigation, with superimposed firing activity of the unit (black line with blue dots), the firing rate map (blue square), and a single unit waveform (corresponding to a claustral place cell). On completion of the recording experiments, positions of recorded cells were estimated by histological analyses (the red circle shows tetrodes in the claustrum). (b) Examples of six claustral place cells recorded over consecutive sessions with changes in the environmental conditions; when the light was eliminated from the environment, a shift of the claustral place field occurred with a L1DL2 angle (c) of about 50°. (d) The measure of the distance between the centre of mass (COM) of each place field in three different conditions (L1, D, and L2) revealed a shift of the place field in the dark. (e) Spatial coherence was statistically different between light and dark conditions, and there were no differences between the first and the last light exposure

We performed recordings (see Section 4 and Figure 1a) in which rats navigated in a square arena while foraging for food pellets. Additionally, we investigated the effects of light/dark transitions to explore how claustral places are modulated by changes in ambient visual inputs (i.e., light or dark during the recording session; Brotons‐Mas et al., 2010). In the light condition, the arena was indirectly lit by four symmetrically positioned spotlights. The animal navigated in a square arena for 30 min; visual inputs changed every 10 min from light (L1) to dark (D) and back to light (L2). At the end of each session, the arena was cleaned to remove odours. The next day (Day 2), rats received a subcutaneous injection of D‐SER (*n* = 6 rats) 30 min before recording. Recordings were repeated at the same time as Day 1 following the same procedure (L1_D‐SER_, D_D‐SER_, and L2_D‐SER_). This protocol was used to measure the place field stability/remapping between different conditions and their modulation by NMDA receptor activity. The D‐SER effect was also assessed via oscillatory power (total power, subgroup, *n* = 3), before and after drug administration.

### 2.1 Claustral place cells remap in darkness

We found place fields were stable between the two sessions in light (before and after dark exposure). When visual inputs were eliminated from the environment, place fields remapped their position consistently and significantly. The measure of the distance between the centre of mass (COM) of each place field in the three different conditions (L1, D, and L2) revealed a shift of the place field from light to dark (L1–D). The place field returned to a similar position in the first light exposure during the last recording in light (L2) (ΔL1–D (cm) = 12.75 ± 2.06, ΔL1–L2 (cm) 10.00 ± 1.92, ΔD–L2 (cm) = 9.44 ± 2.26, mean ± SEM, Figure 1d). A consistent angle was formed by the three place fields (L1, D, and L2) in the arena (Figure 1b). We used the centre of mass of the place fields (*x* and *y* coordinates) to measure potential differences between the three conditions (see Section 4 for details). Notably, we found claustral place cells consistently remapped by about 50° in darkness (L1DL2 angle [degrees]: 47.84 ± 6.82, *n* = 19, mean ± SEM, Figure 1c), compared with light conditions.

Overall, our results show that when visual inputs are removed the claustral place map moves to a new, constant, location in the arena in when the rat is navigating in darkness.

### 2.2 NMDA receptor modulation sharply changes claustral place cell remapping

**FIGURE 2:**
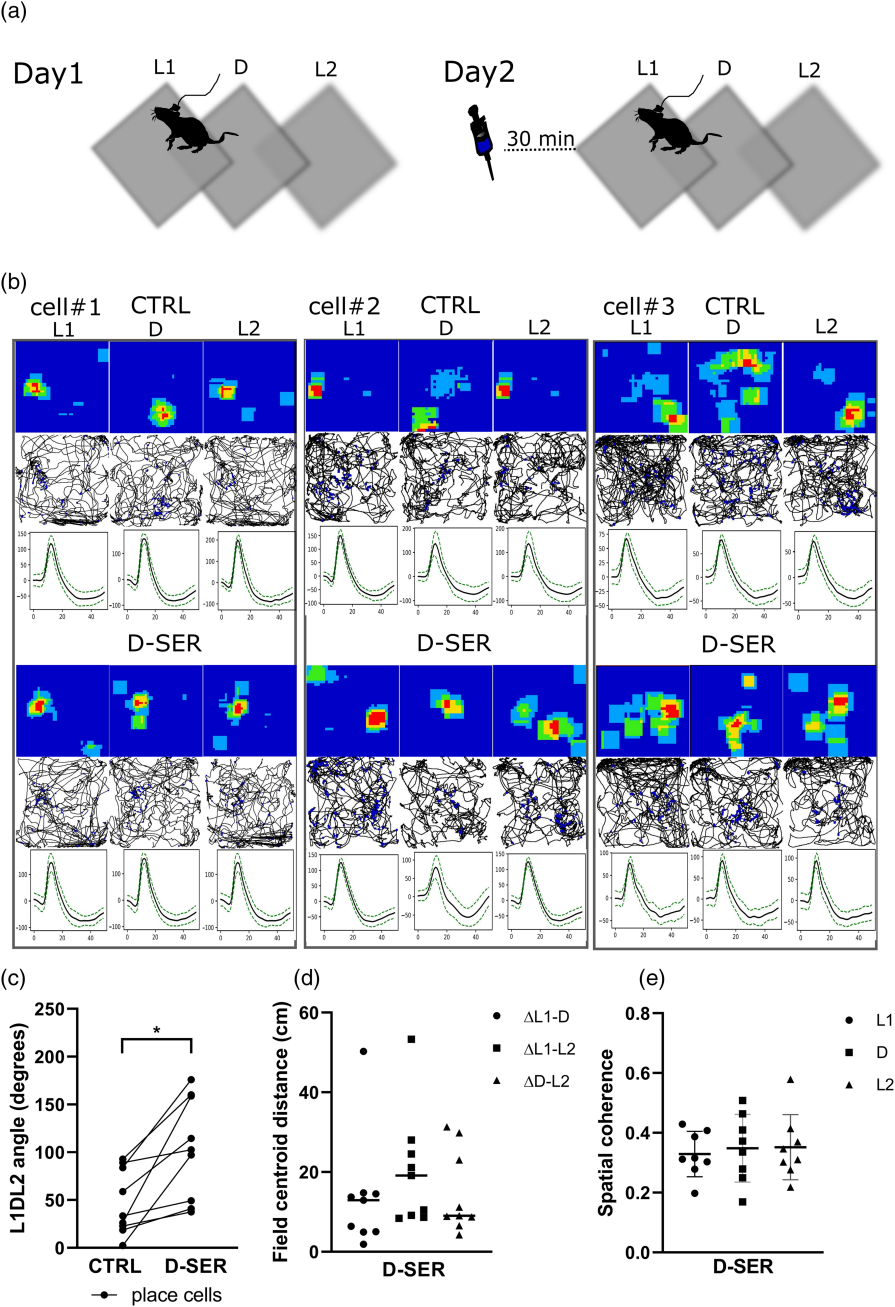
NMDA receptor activation reduces claustral place cells remapping. (a) Day 2 replicated Day 1 (but included subcutaneous administration of D‐SER; 10 mg/kg). (b) Example of three claustral place cells in control, and following D‐SER administration; place cell remapping in dark was reduced following D‐SER administration. (c) L1DL2 angle and (d) distance increased following D‐SER administration. (e) Spatial coherence was not statistically different between light and dark following D‐SER administration

We tested whether the modulation of NMDA receptor activity decreased remapping in the dark. First, 30 min before the recording session of Day 2, we subcutaneously injected the NMDA receptor co‐agonist D‐SER (see Section 4). The experimental procedure of Day 2 (day in which rats received the drug) was the same as Day 1 (L1/D/L2), except for the drug pre‐injection administration (during a time window where the drug shows its highest peak of activity; Figure 2a). We found again the claustral place cells consistently remapped by about 50 degrees in darkness, but that the angle was significantly increased following D‐SER injection in each cell recorded (L1DL2 angle _CTRL_ [degrees] = 47.57 ± 11.39 vs. L1DL2 angle _D SER_ = 104.13 ± 17.82, *p* = 0.003, *n* = 9, mean ± SEM, two‐tailed *t* test for paired two samples for means, Figure 2c). The increase in the angle indicates that the three place fields were formed at a similar location. In D‐SER treated rats, the measure of the difference in terms of distance between the place field CoM in L1, D, and L2 revealed the three place fields at a similar location (ΔL1–D (cm) = 13.82 ± 4.84, ΔL1–L2 (cm) 20.30 ± 4.81, ΔD–L2 (cm) = 14.74 ± 3.47, mean ± SEM, Figure 2d).

We measured spatial (Skaggs) information content in controls, and following drug administration, in each condition. Consistent with Jankowski and OMara (2015), we found that the spatial information content was significantly higher in the dark (D_CTRL_ = 3.09 ± 0.32, mean ± SEM), compared with the preceding recording in light (L1_CTRL_ = 2.54 ± 0.29, *p* ≤ 0.01, mean ± SEM, two‐tailed *t* test paired). Contrarily, we found a difference in spatial information content between the recordings in the dark (D_CTRL_ = 3.09 ± 0.32, mean ± SEM) and the following recordings in the light (L2_CTRL_ = 2.36 ± 0.34, *p* < 0.01, mean ± SEM, two‐tailed *t* test paired). There were no significant differences in spatial information content between the first and the last recording in light (L1_CTRL_ vs. L2_CTRL_ *p* = 0.06, two‐tailed *t* test). Interestingly, there were no significant differences in spatial information content between L1, D, and L2 following D‐SER administration (L1_D‐SER_ = 3.17 ± 0.43 vs. D_D‐SER_ = 3.14 ± 0.29, *p* = 0.93; D_D‐SER_ = 3.14 ± 0.29 vs. L2_D‐SER_ = 3.12 ± 0.31, *p* = 0.92; L1_D‐SER_ = 3.17 ± 0.43 vs. L2_D‐SER_ = 3.12 ± 0.31, *p* = 0.92, *n* = 9, mean ± SEM, two‐tailed *t* test paired).

The measure of spatial coherence across conditions revealed a significant difference between the first light and dark (L1_CTRL_ = 0.30 ± 0.05 vs. D_CTRL_ = 0.39 ± 0.04, *p* = 0.05, Figure 1e) and between dark and the last light (D_CTRL_ = 0.39 ± 0.04 vs. L2_CTRL_ = 0.31 ± 0.03, *p* ≤ 0.01). There was no difference in spatial coherence between first and last light exposure (L1_CTRL_ = 0.30 ± 0.04 vs. L2_CTRL_ = 0.31 ± 0.03, *p* = 0.73, mean ± SEM, two‐tailed *t* test paired, Figure 1e). The difference in the spatial coherence disappeared following D‐SER injection between the first light and dark (L1_D‐SER_ = 0.32 ± 0.02 vs. D_D‐SER_ = 0.35 ± 0.04, *p* = 0.52, *n* = 9, mean ± SEM, two‐tailed *t* test paired, Figure 2e) and between dark and the last light (D_D‐SER_ = 0.35 ± 0.04 vs. L2_D‐SER_ = 0.35 ± 0.04, *p* = 0.94, *n* = 9, mean ± SEM, two‐tailed *t* test paired). D‐SER administration had no effect in the spatial coherence similarity between the first and last light (L1_D‐SER_ = 0.32 ± 0.02 vs. L2_D‐SER_ = 0.35 ± 0.04, *p* = 0.42, *n* = 9, mean ± SEM, two‐tailed *t* test paired).

### 2.3 Single unit firing properties are modulated by NMDA receptor activation

**FIGURE 3:**
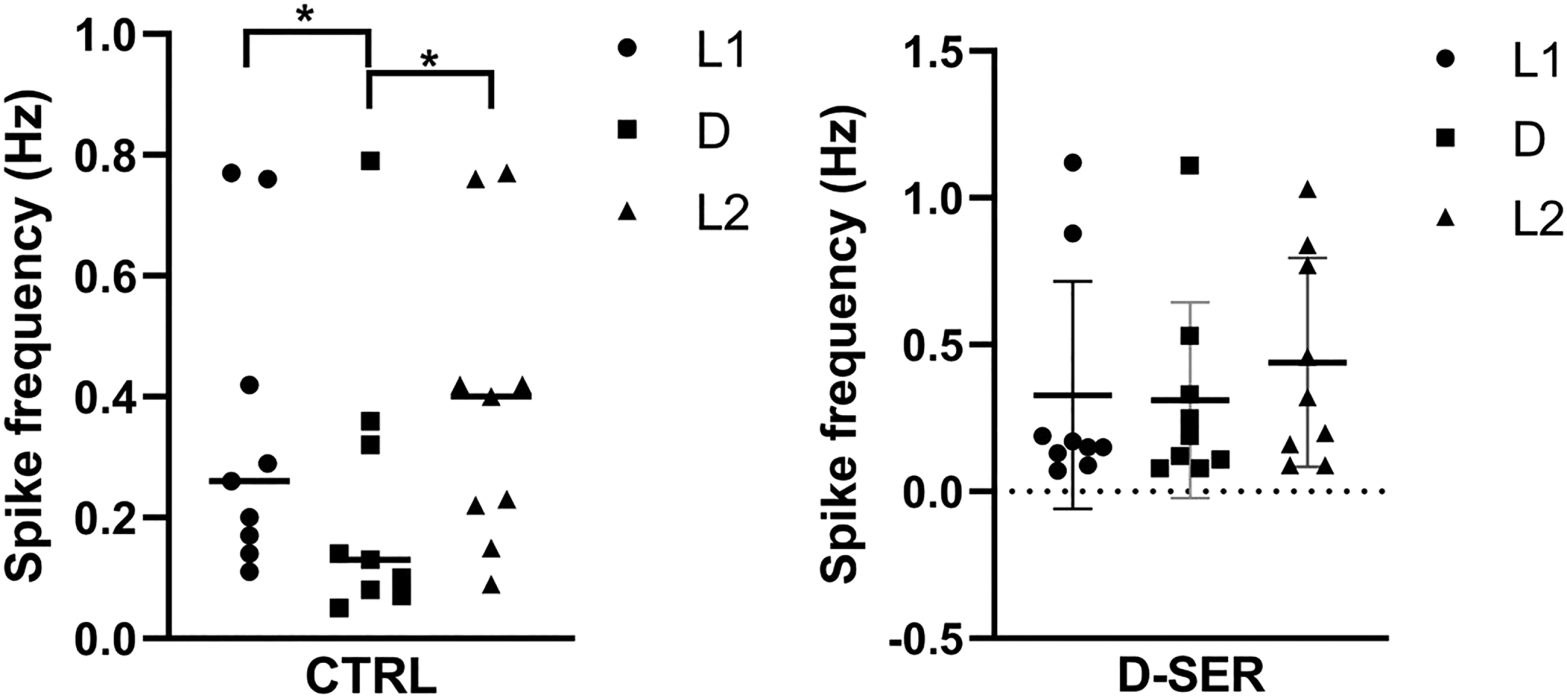
Claustral place field remapping underlies a change of firing. (a) The exposure to darkness significantly decreased claustral place cell firing. (b) D‐SER eliminated differences in the spike frequency between light and dark

We next investigated whether the drug effect on place field remapping in darkness underlies the firing changes of single place cells in the claustrum. To this end, we measured both the action potential and firing properties in controls and following drug administration. Previous studies demonstrated that place cell firing properties change between light and dark in the hippocampus (Zhang et al., 2014). However, we observed no statistically significant differences in the control recordings between L1/D/L2 transition for spike amplitude (L1_CTRL_ [μV] = 134.90 ± 19.76 vs. D_CTRL_ = 153.38 ± 21.73, *p* = 0.22, *n* = 9; D_CTRL_ = 153.38 ± 21.73 vs. L2_CTRL_ = 122.51 ± 12.46, *p* = 0.06, *n* = 9; L1_CTRL_ = 134.90 ± 19.76 vs. L2_CTRL_ = 122.51 ± 12.46, *p* = 0.31, *n* = 9, mean ± SEM, two‐tailed *t* test paired) and spike width (L1_CTRL_ [μs] = 249.20 ± 9.01 vs. D_CTRL_ = 245.48 ± 19.04, *p* = 0.82, *n* = 9; D_CTRL_ = 245.48 ± 19.04 vs. L2_CTRL_ = 257.11 ± 14.55, *p* = 0.34, *n* = 9; L1_CTRL_ = 249.20 ± 9.01 vs. L2_CTRL_ = 257.11 ± 14.55, *p* = 0.46, *n* = 9, mean ± SEM, two‐tailed *t* test paired). Interestingly, spike frequency was significantly lower in the dark, compared with the preceding recording in light (L1_CTRL_ [Hz] = 0.35 ± 0.09 vs. D_CTRL_ = 0.23 ± 0.09, *p* = 0.04, *n* = 9, mean ± SEM, two‐tailed *t* test paired, Figure 3a), as well as the following recording in light (D_CTRL_ [Hz] = 0.23 ± 0.09 vs. L2_CTRL_ = 0.39 ± 0.08, *p* = 0.02, *n* = 9, mean ± SEM, two‐tailed *t* test paired). There were no significant differences in the spike frequency between the first and final recording in light (L1_CTRL_ [Hz] = 0.35 ± 0.09 vs. L2_CTRL_ = 0.39 ± 0.08, *p* = 0.08, *n* = 9, mean ± SEM, two‐tailed *t* test paired).

These findings raise the question: Is the decrease in remapping by D‐SER caused by a change of firing? To address this issue, we analysed spike frequency following D‐SER injection. Notably, D‐SER removed the statistical difference between L1/D/L2 in spike frequency (L1_D‐SER_ (Hz) = 0.33 ± 0.13 vs. D_D‐SER_ = 0.31 ± 0.11, *p* = 0.75, *n* = 9, mean ± SEM, two‐tailed *t* test paired, Figure 3b; D_D‐SER_ = 0.31 ± 0.11 vs. L2_D‐SER_ = 0.44 ± 0.12, *p* = 0.13, *n* = 9, mean ± SEM, two‐tailed *t* test paired; L1_D‐SER_ = 0.33 ± 0.13 vs. L2_D‐SER_ = 0.44 ± 0.12, *p* = 0.27, *n* = 9, mean ± SEM, two‐tailed *t* test paired, Figure 3b), suggesting the decrease in place field remapping following D‐SER injection might result from an increase in place cell firing in darkness.

### 2.4 Visual manipulations do not interfere with bursting cell activity

We next investigated whether the influence of the visual input elimination was extended to other cells observed in the claustrum. We found that the 18.37% of the cell population in the claustrum were bursting cells without spatially‐related firing. Following their characterization (Table 1), we analysed spike properties and firing rate of bursting cells over the L1/D/L2 sequence. There were no differences in the spike width over the three conditions (L1 [μs] = 253.00 ± 84.33 vs. D = 254.13 ± 84.71, *p* = 0.78, *n* = 9; D = 254.13 ± 84.71 vs. L2 = 251.94 ± 83.98, *p* = 0.58, *n* = 9; L1 = 253.00 ± 84.33 vs. L2 = 251.94 ± 83.98, *p* = 0.79, *n* = 9, mean ± SEM, two‐tailed *t* test paired). Similarly, no statistically significant differences were observed in the spike amplitude over the three conditions (L1 [μV] = 126.04 ± 42.01 vs. D = 120.31 ± 40.10, *p* = 0.23, *n* = 9; D = 120.31 ± 40.10 vs. L2 = 124.81 ± 41.60, *p* = 0.28, *n* = 9; L1 = 126.04 ± 42.01 vs. L2 = 124.81 ± 41.60, *p* = 0.69, *n* = 9, mean ± SEM, two‐tailed *t* test paired). The light/dark contrasts had no effect on the spike frequency of bursting cells (L1 [Hz] = 3.06 ± 1.02 vs. D = 3.01 ± 1.00, *p* = 0.93, *n* = 9; D = 3.01 ± 1.00 vs. L2 = 3.03 ± 1.00, *p* = 0.97, *n* = 9; L1 = 3.06 ± 1.02 vs. L2 = 3.03 ± 1.00, *p* = 0.91, *n* = 9, mean ± SEM, two‐tailed *t* test paired). Finally, we measured the total number of burst events for each cell and found visual stimuli did not affect bursting activity (L1 = 60.33 ± 20.11 vs. D = 45.22 ± 15.07, *p* = 0.45, *n* = 9; D = 45.22 ± 15.07 vs. L2 = 65.38 ± 23.11, *p* = 0.29, *n* = 9; L1 = 60.33 ± 20.11 vs. L2 = 65.38 ± 23.11, *p* = 0.42, *n* = 9, mean ± SEM, two‐tailed *t* test paired). Thus, the manipulation of visual stimuli in the environment does not affect spike and firing properties of claustral bursting cells.

### 2.5 Locomotor activity is not influenced by D‐SER administration

Place cell firing rate is positively correlated with the animals' running speed. To determine whether the firing rate modulation by D‐SER correlates with changes in running speed, we measured average running speeds before and after drug administration (L1_CTRL_ vs. L1_D‐SER_). Our data suggest that D‐SER did not influence the animal's behaviour during navigation (L1_CTRL_ [cm/s] = 10.05 ± 4.10 vs. L1_D‐SER_ = 9.47 ± 3.87, *n* rats = 6, *p* = 0.71, n.s. mean ± SEM, two‐tailed *t* test paired), suggesting D‐SER did not affect running speed.

### 2.6 Claustral theta oscillations are not affected by changes to visual inputs

**FIGURE 4:**
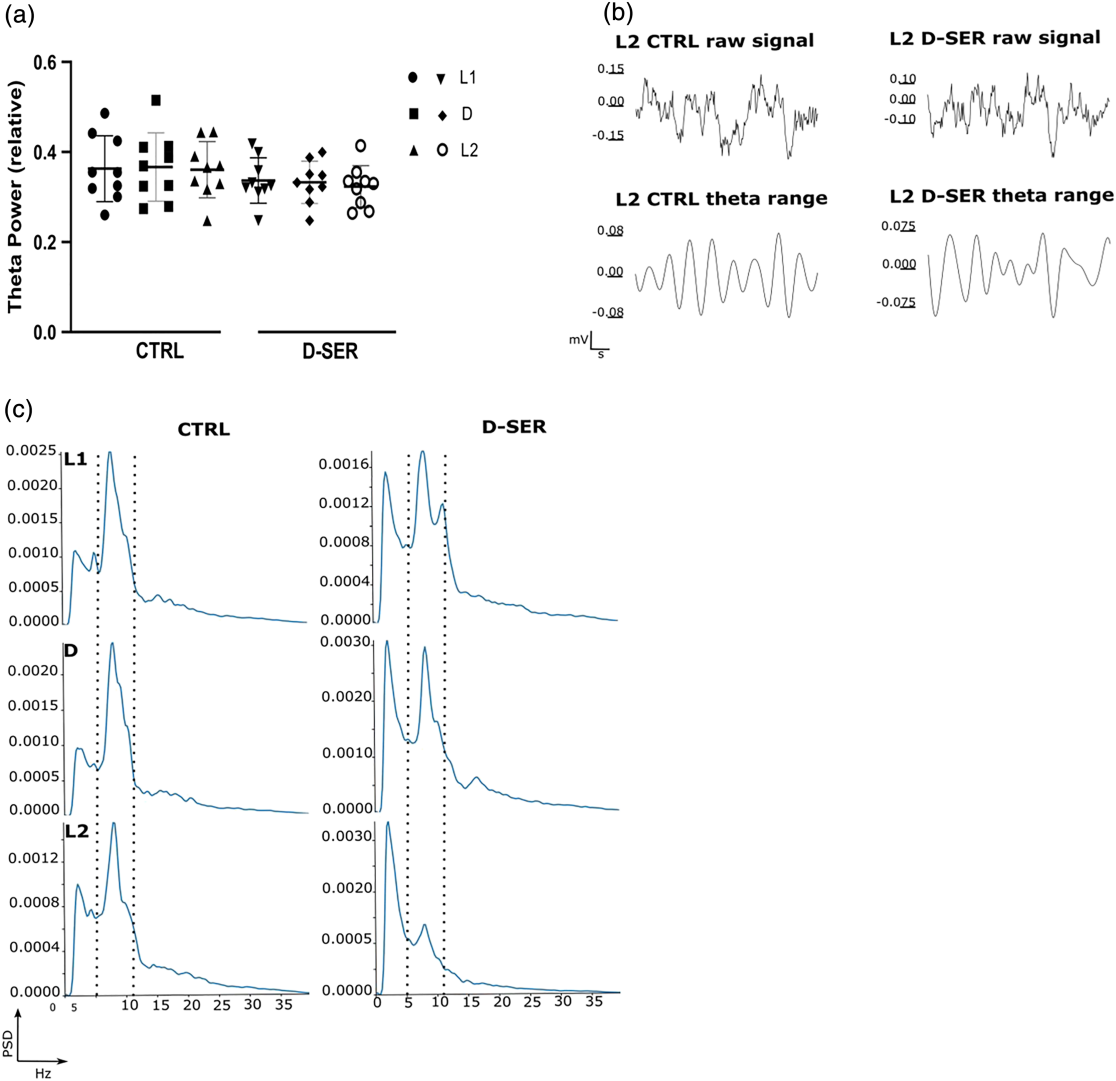
Theta oscillations following light and NMDAR manipulation. (a) Theta power did not change following visual manipulation; D‐SER administration did not affect theta power oscillations along the L1/D/L2 sequence. (b) Example of raw LFP traces and in the theta range (1 s selected from 10 min of LFP recording) in control and following D‐SER administration during recordings in L2. (c) Periodogram in L1/D/L2 in control and following D‐SER administration (theta range is highlighted by the black dashed)

To determine how theta oscillations are modulated by dark exposure, we measured the relative power of theta oscillations over the L1/D/L2 sequence, and compared how NMDA receptor activation by the co‐agonist D‐SER influences the claustral local circuit and the neuronal response to light/dark transition. Specifically, we compared theta oscillations for control (Day 1), following D‐SER administration (Day 2), and across the 2 days of recording (Day 1 vs. Day 2). Visual input manipulations had no effect on oscillations. Likewise, D‐SER administration did not affect the relative power of theta oscillations over the L1/D/L2 sequence. (L1_CTRL_ = 0.36 ± 0.02 vs. D_CTRL_ = 0.37 ± 0.03, *p* = 0.72, *n* = 9; D_CTRL_ = 0.37 ± 0.03 vs. L2_CTRL_ = 0.36 ± 0.02, *p* = 0.63, *n* = 9; L1_CTRL_ = 0.36 ± 0.02 vs. L2_CTRL_ = 0.36 ± 0.02, *p* = 0.87, *n* = 9, mean ± SEM, two‐tailed *t* test paired, Figure 4a; L1_D‐SER_ = 0.34 ± 0.02 vs. D_D‐SER_ = 0.33 ± 0.02, *p* = 0.53, *n* = 9; D_D‐SER_ = 0.33 ± 0.02 vs. L2_D‐SER_ = 0.32 ± 0.02, *p* = 0.27, *n* = 9; L1_D‐SER_ = 0.34 ± 0.02 vs. L2_D‐SER_ = 0.32 ± 0.02, *p* = 0.21, *n* = 9, mean ± SEM, two‐tailed *t* test paired, Figure 4a). Although a visible theta oscillation reduction was found by comparing each subset of visual stimuli input before and after drug administration, the overall difference was not statistically significant (L1_CTRL_ = 0.36 ± 0.02 vs. L1_D‐SER_ = 0.34 ± 0.02, *p* = 0.074, *n* = 9; D_CTRL_ = 0.37 ± 0.03 vs. D_D‐SER_ = 0.33 ± 0.02, *p* = 0.074, *n* = 9; L2_CTRL_ = 0.36 ± 0.02 vs. L2_D‐SER_ = 0.32 ± 0.02, *p* = 0.055, *n* = 9, mean ± SEM, Wilcoxon matched‐pairs signed rank test). These results suggest that the theta oscillations do not correlate with claustral place cell remapping in darkness.

**FIGURE 5:**
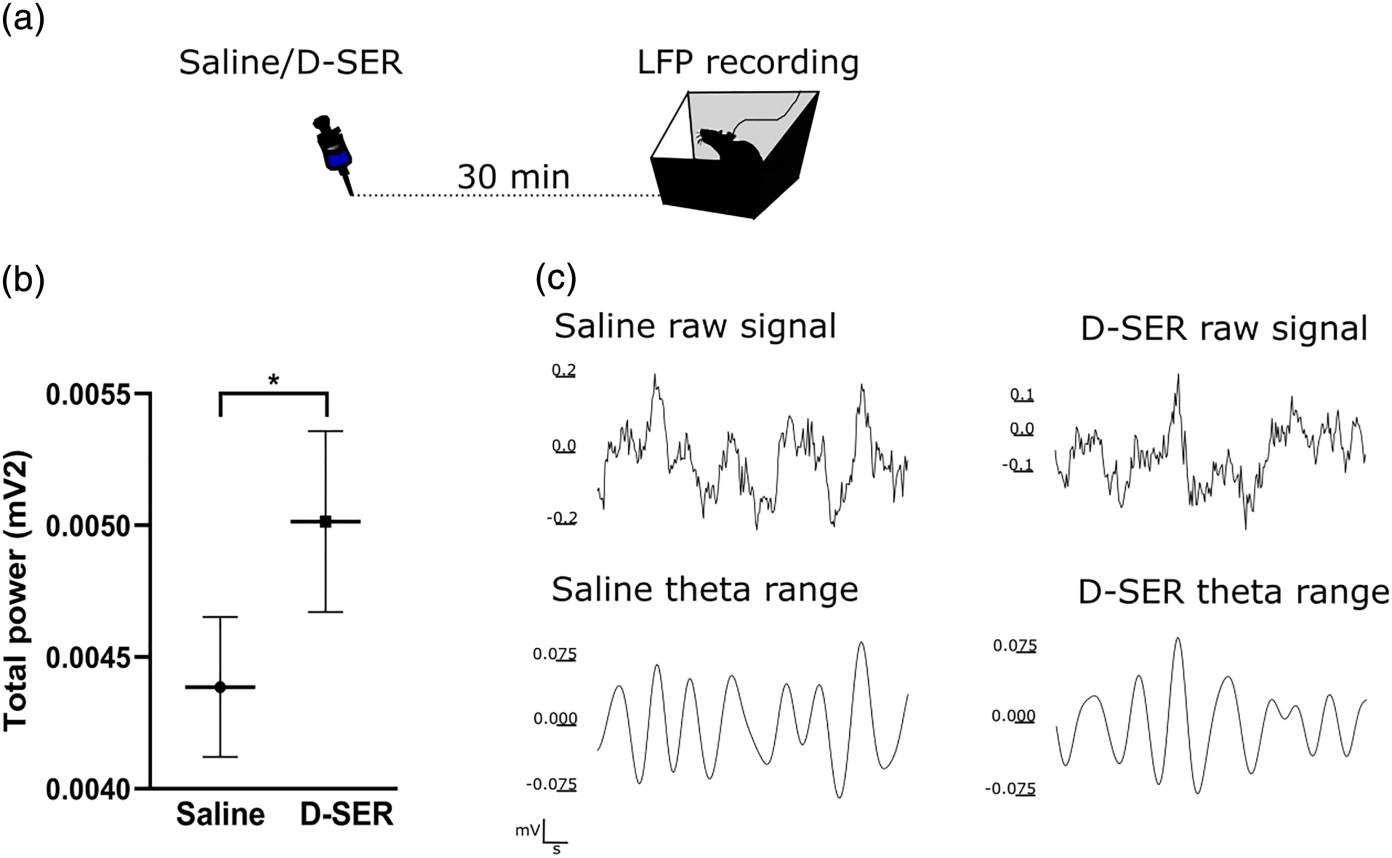
Total power increases following D‐SER administration. (a) LFPs were recorded following a subcutaneous injection of normal saline (Day 1) or D‐SER (Day 2) in a box (24h × 38w × 38l cm). (b) D‐SER significantly increases the total power in the claustrum. (c) Representative traces (1 s segments extracted from 10 min recording) of the total power in saline (top) and following D‐SER administration (bottom)

Then, to determine how effectively D‐SER influences the local claustral network, we examined total power following drug administration. The administration of D‐SER significantly increased the total power in the claustrum (total power saline [μV^2^] = 4710.67 ± 269.51 vs. total power D‐SER [μV^2^] = 5325.92 ± 339.12, *p* < 0.01, *n* LFP traces = 24 [*n* = 4 LFP traces for each rat, *n* of rats = 3, *n* sessions = 2 saline vs. 2 D‐SER], mean ± SEM, two‐tailed *t* test paired, Figure 5). These results show that the dose of D‐SER used for place cell recordings effectively activated NMDAR receptors, increasing claustral neuronal firing.

### 2.7 Claustral delta and theta oscillations change from light to dark

The constant, reliable angle formed by remapping claustral place fields in darkness led us to hypothesize that remapping arose from a loss of inputs from visual cortices to the claustrum, and therefore a loss of visual calibration to support place field stability. The claustrum receives dense ipsilateral inputs from visual cortices and projects back to these areas. We therefore recorded oscillatory activity in secondary visual cortex and claustrum during navigation in light and dark.

**FIGURE 6:**
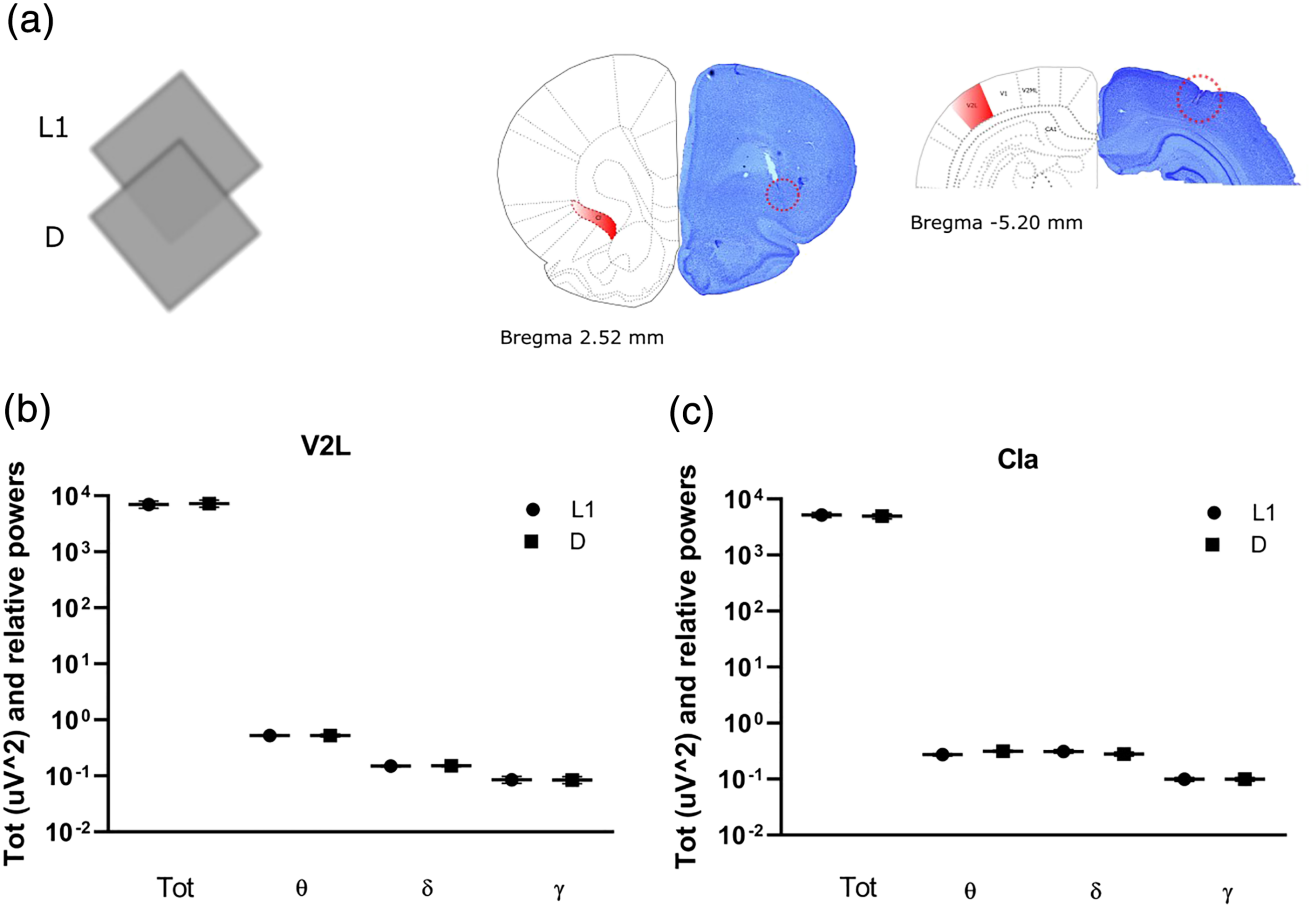
Claustral delta and theta oscillations change from light to dark. (a) The animals navigated in a square arena which changed from light (L1) to dark (D); tetrodes and electrodes positions were estimated with histological analyses (the red circle shows tetrodes in the claustrum (left) and electrodes in V2L (right). Total and relative powers in V2L (b) and claustrum (c) from L1 to D

The first question was to investigate whether the protocol that showed us the remapping caused changes in both visual and claustral oscillatory activity. Following tetrode implantation in these two brain areas, animals navigated in the square arena moving from light (L1) to dark (D) (Figure 6a) while the local field potential was simultaneously recorded. The total power of raw LFP oscillations in the V2L (lateral secondary visual area) cortex was different between light and dark (L1_tot_ [μV^2^] = 7061.288 ± 1044.802 vs. D_tot_ [μV^2^] = 7326.231 ± 1016.661, *p* = 0.031, *n* samples = 6, Figure 6b; Wilcoxon signed‐rank test). The V2L relative powers in the θ, δ, and γ bands were not different between light and dark (L1_θ_ = 0.526 ± 0.014 vs. D_θ_ = 0.530 ± 0.032, *p* = 1.000; L1_δ_ = 0.150 ± 0.006 vs. D_δ_ = 0.152 ± 0.006, *p* = 1.000; L1_γ_ = 0.086 ± 0.012 vs. D_γ_ = 0.085 ± 0.012, *p* = 0.563; Wilcoxon signed‐rank test). In the claustrum, the total power of raw LFP oscillations and the relative power of γ oscillations were not different between light and dark, while θ and δ oscillations substantially changed (L1_tot_ [μV^2^] = 5235.302 ± 534.739 vs. D_tot_ [μV^2^] = 4971.775 ± 508.535, *p* = 0.084, *n* samples = 10; L1_γ_ = 0.099 ± 0.008 vs. D_γ_ = 0.099 ± 0.007, *p* = 0.695; L1_θ_ = 0.274 ± 0.014 vs. D_θ_ = 0.314 ± 0.015, *p* = 0.002; L1_δ_ = 0.312 ± 0.023 vs. D_δ_ = 0.280 ± 0.022, *p* = 0.002, Figure 6c; Wilcoxon signed‐rank test). These results suggest that the switch from light to dark modulated the oscillatory activity of both visual cortex and claustrum leading to place cells remapping.

## 3 DISCUSSION

Here, we explored how claustral place cells remap when visual inputs are eliminated from the environment, finding they remap by a constant angle; this remapping is likely to be NMDA‐receptor‐dependent. Furthermore, eliminating visual inputs enhances theta‐ and reduces delta‐band oscillatory activity in the claustrum, while increasing simultaneously recorded visual cortical activity. We conclude that, like the hippocampus, claustral place field remapping might be mediated by NMDA receptor activity, and is modulated by visual cortical inputs. We also investigated claustral unit firing changes to explore the mechanism underlying claustral place field remapping in darkness. We established there was an absence of rate remapping between the two light conditions (i.e., before and following the dark condition). Intriguingly, there was a reduction in firing rates when visual inputs were eliminated from the environment, accompanied by place remapping in the dark.

Several lines of evidence suggest spatial maps (including their stability and expression) are influenced by plasticity dependent mechanisms. NMDA receptor‐dependent synaptic plasticity is necessary for establishing stable neuronal representations in the hippocampus (Kentros et al., 1998; Shapiro, 2001). Indeed, Shapiro (2001) concluded NMDA receptor antagonists prevent the establishment of stable place fields in the hippocampus. Furthermore, Kentros et al. (1998) observed that blocking NMDA receptors abolished the long‐term stability of place field remapping in the hippocampus. Thus, to explore the mechanisms underlying claustral place field remapping in darkness, we administered the NMDA receptor co‐agonist D‐SER before performing the light/dark/light recording. In addition to glutamate, NMDA receptor activation needs the binding of a co‐agonist at the GluN1 subunit (Oliet & Mothet, 2009). Recently, it has emerged that only D‐SER gates NMDA receptors at the synaptic level, whereas extra‐synaptic receptors are gated by glycine (Papouin et al., 2012; Sullivan & Miller, 2012). Furthermore, D‐SER gates NMDA receptors only in the mature brain, whereas glycine gates NMDA receptor activity in the immature brain (Ferreira et al., 2017). In our study, we used adult rats with a weight of about 300–350 g. For these reasons, we chose D‐SER as the more effectively co‐agonist of NMDA receptors, with an expectation of a reduction in place field remapping. Accordingly, we found D‐SER strongly reduced remapping (Day 2 vs. Day 1). Moreover, after D‐SER administration, we observed an increase in neuronal firing in darkness. Indeed, NMDA receptor activation increased firing rates in claustral place cells when the rats navigated in darkness. We conclude that D‐SER affects place fields in darkness, perhaps by increasing claustral place cell firing.

Next, to assess the role of NMDA receptor co‐activation in the local circuit, we investigated claustral oscillations following drug administration. Measurement of theta power (oscillations active during exploratory behaviour) revealed place field remapping in darkness was not driven by changes in the local network in the claustrum. Theta waves, which are present during various types of locomotor activities, have been assumed to be generated by the activation of NMDA receptors. Specifically, a combination of NMDA receptor blockers abolished all theta activity in the hippocampus (Buzsaki, 2002). Here, we observed theta power was not modulated by NMDA receptor activation in the claustrum.

The constant angle formed in darkness by claustral place cells and its substantial reduction following D‐SER administration led us to hypothesize that synaptic plasticity might mask an underestimated input to the claustrum. Claustral place cell responses may have visual cortical inputs; interestingly, transitioning from light to dark resulted in changes in activity in secondary visual cortex, and increased theta‐ while reduced delta‐band claustral oscillations. Delta oscillations may support attentional dynamics (Harmony, 2013); perhaps claustral remapping involves processes related to attention, rather than direct visual inputs.

Overall, our findings show that NMDA receptor activation stabilizes claustral place fields in darkness, perhaps by increasing the strength of synaptic transmission. Our findings of differential visual cortical and claustral activity during the light/dark protocol, expand on previous findings suggesting that claustral place cell remapping might be due to attentional dynamics.

## 4 MATERIAL AND METHODS

### 4.1 Subjects

A total of 13 male Lister‐hooded rats (Envigo, United Kingdom) weighing 300–350 g were used. Upon arrival, they were housed two per cage and kept in a temperature‐controlled laminar airflow unit with a 12/12 h light/dark cycle beginning at 8:00 AM with ad libitum access to food and water. They were handled daily for 10 days before electrode implantation. Rats were observed and handled during a recovery period of 10 days following electrode implantation. Before starting with electrophysiological recordings, animals underwent a food deprivation procedure until they reached 85% of free‐feeding body weight; they were maintained at this weight during the entire study. In three of the animals, a preliminary screening was performed using a single recording of 10 min where rats navigated in a square arena to ensure the presence of place cells in the claustrum. In 13 animals, we performed electrophysiological recordings following microdrive (Axona, Ltd, UK) implantation in the claustrum using a light/dark/light protocol. In six animals, D‐SER was systemically administrated. LFP recordings were performed in three animals to determine how effectively the 10 mg/kg dose D‐SER influences the local claustral network. Finally, in three animals, we performed electrophysiological recordings following microdrive implantation in both the claustrum and secondary visual cortex.

### 4.2 Compliance with ethical standards

All experimental procedures were in accordance with the ethical, welfare, legal, and other requirements of the Healthy Products Regulatory Authority regulations and were compliant with the Health Products Regulatory Authority (IrishMedicines Board Acts, 1995 and 2006) and European Union directives on Animal Experimentation (86/609/EEC and Part 8 of the EU Regulations 2012, SI 543). All experimental procedures were approved by the Comparative Medicine/Bioresources Ethics Committee, Trinity College Dublin, Ireland, before conduct and were conducted in accordance with LAST Ireland and international guidelines of good practice.

### 4.3 Behavioural testing

#### 4.3.1 Place cells recordings in light/dark

The experimenter entered the recording room at the start and the end of each sequence of a session to clean rat urine traces in the arena, for sugar pellet throwing and to disentangle the recording cable. Electrophysiological recordings were performed from rats navigating an 80 × 80 cm square and black arena (see Figure 1a). The arena was in the middle of an opaque, black‐curtained, square enclosure. The curtains were open/closed during recordings in light/dark respectively. All the equipment and devices (including the recording setup and computers) of the room were covered with black bags to reduce visual cues. During the recording in light the apparatus was indirectly lit by four symmetrically positioned spotlights. During foraging, a small amount of pellet (TestDiet™, 5 TUL, USA) was thrown in the arena at random locations.

### 4.4 LFP spectra recording

After a recovery period from tetrode implantation in the claustrum, the head stage was connected to the microdrive for recordings. Three rats were subcutaneously injected with normal saline (Day 1, NaCl, 0.9% 10 mg/kg) and D‐SER (Day 2, 10 mg/kg, Sigma‐Aldrich Ireland) and placed in a box (24h × 38w × 38l cm). The LFP recording started 30 min following the substance administration (Figure 5a). LFPs were recorded for 30 min during which sugar pellets were thrown in the box every 5 min. The experimenter was outside the room for the entire recording, except every 5 min for pellet throwing. The oscillatory activity from the secondary visual cortex and claustrum was simultaneously recorded during light/dark while animals navigated in a square arena.

### 4.5 Electrode implantation

Following the acclimation period, animals underwent surgery for electrode implantation 25 μm above the claustrum to avoid tissue damages. Rats were implanted with bundles of eight tetrodes of ø 25 μm platinum‐iridium wires with impedance 150–350 KΩ (California Fine Wire Ltd., CA, USA). Microdrives were built using eight tetrodes and implanted after craniotomy in the caudal part of the anterior claustrum, according to the atlas coordinates (Paxinos and Watson, 5th Edition). The claustral coordinates used relative to bregma were as follows: anterior‐posterior (AP) +2.52 mm, medial‐lateral (ML) ±2.00 mm, dorso‐ventral (DV) −4.6 mm from top of the cortex, at an angle of 13°. Coordinates for stereotaxic implantation of a bipolar electrode (Stainless steel wire, SS‐5T 127 μm, Science Products GmbH, Hofheim, DE) in secondary visual cortex relative to bregma were as follows: anterior‐posterior (AP) −5.28 mm, medial‐lateral (ML) ±4.00 mm, dorso‐ventral (DV) −1.4 mm from top of the cortex, at an angle of 24.8°. The bipolar electrode was connected to two microdrive electrodes.

### 4.6 Screening and recording

After the recovery period, tetrodes were lowered about 25 μm to the upper layer of the claustrum. A delay of 24 h was interposed between this stage and the screening sessions. During screening sessions, a cable connecting the recording system to the head‐stage (Axona Ltd., UK) was plugged into the microdrive (Axona Ltd., UK). During rat navigation, a screening/recording was performed by using Axona system and software DacqUSB (Axona Ltd., UK). Tetrodes were lowered slowly through the brain (25 μm/day) until single unit signals were considered of sufficient amplitude, with spike properties typical of place cells (Table 1) and displaying a place field. Signals were amplified between 3000 and 12,000 and bandpass filtered between 380 Hz and 7 kHz for single‐unit detection. A camera was located above the arena allowing tracking of the animal's head position. The animal's path was visualized and recorded by the software DacqUSB (Axona Ltd., UK).

### 4.7 Data analysis

#### 4.7.1 Spike units

Graphical, cluster‐cutting, Tint software (Axona Ltd., UK) was used to cluster‐cut the waveforms selected. Place cells were considered as those cells with specific electrophysiological properties (ref., Table 1) that visually and statistically showed a place field. Based on their activity, unit identification involved further criteria: All included as place cells had to have a spatial information content (Skaggs et al., 1993) index ≥ 0.5, a spatial coherence ≥ 0.25, and a mean firing rate ≥ 0.25. The spatial path of the subject and the spike train were used to produce a locational firing rate map. A percentage (16%) of the recorded place cells disappeared after the first session (L1), possibly due to positional instability of the electrodes and/or the cell. In this case, the cell was counted as a place cell according to its properties, but without the following D/L2 sessions. Statistical analyses for mean spike amplitude, mean spike‐width, mean firing frequency, mean inter‐spike interval (ISI), spatial information content (Skaggs) and spatial coherence (mean ± SEM) was performed (Table 1). The spike amplitude (μV) was measured by the difference between the first negative peak and first positive peak, spike‐width (μs) was taken at 25% of the spike amplitude; firing frequency was considered as the number of spikes per second (Hz), the ISI (ms) was represented by the time between subsequent action potentials. Spatial information content represented how much information the firing rate of a cell contained about the spatial location of the rat. It was calculated using the methods from (Skaggs et al., 1993). Spatial coherence represented the correlation between the raw firing map and smoothed firing map. The measure of the total burst as the total number of burst events in a cell was used for bursting cells. NeuroChaT (Islam et al., 2019) was used to analyse the interspike interval (ISI), frequency and cell bursting properties from the spike train of bursting cells. The rhythmic effect of the cell was amplified by the autocorrelation measure of ISI histogram. Indeed, bursting cells were discriminated among other cells on account of their fast firing profile, a higher firing frequency and a lower ISI compared to place cells (Table 1), an ISI of minimum 6 ms, a minimum interburst interval (IBI) of 50 ms. Additionally, the total number of bursts during a recording session, the number of spikes in the bursting cluster, number of spikes per burst, burst duration and propensity to burst were examined.

#### 4.7.2 Local field potential

LFP traces obtained from claustrum were referenced to the animal ground, and power was calculated using a random channel for each tetrode. Relative theta power (theta/total) was calculated for each session (L1, D, and L2) by calculating Welch's periodogram for the LFP signal and then integrating bands of the periodogram (5–11 Hz for theta), using Simpson's method. Total power was calculated by integrating the periodogram between 1.5 and 90 Hz. This range was chosen to eliminate very low and very high frequency noise present in the LFP recording. Relative powers in these studies were calculated by dividing the power in a band giving a measure of how much of the overall LFP activity is accounted for by this frequency band. The range of relative powers was 1.5–4 Hz for delta, 5–11 Hz for theta, and 30–90 Hz for gamma oscillations.

#### 4.7.3 Spatial firing stability

To assess whether place cells had a stable place field between successive sessions, the equation L1DL2 (angle) was used to assess the measure of the angle (degrees) between the place field in darkness (D) and the two place fields in light (L1 and L2) using the COM of each place field:

$$L1\mathrm{DL}2\;\left(\text{angle}\right)=\cos^{-1}\;\frac{\mathrm{DL}1.\mathrm{DL}2}{\left||\mathrm{DL}1|\right|*\|\mathrm{DL}2\|}$$

where DL1 and DL2 (numerators) are the vectors from the place field centroid in D to the place field centroid in L1 and from the place field centroid in D to the place field centroid in L2, respectively. DL1.DL2 is the dot product of the vectors and it is divided by the product between the norm of the vectors DL1 and DL2. The equation below was used to measure the difference in terms of distance (cm) between the place field centroids (*x* and *y* coordinates) in L1 and D, L1 and L2, and D and L2. The difference (cm) allowed to measure the shift in the place field between light and dark in control and following drug injection.

$$∆L1-D=\sqrt{{\left(\mathrm{yD}-\mathrm{yL}1\right)}^{2}+{\left(\mathrm{xD}-\mathrm{xL}1\right)}^{2}}$$

where *x* and *y* are the coordinates of the place field centroid, and the equation represents the distance formula.

#### 4.7.4 Position and speed estimation

DacqTrack (Axona, Ltd, UK) includes various methods for tracking the *x* and *y* coordinates of a moving target within the image. During L1/D/L2 and LFP recordings, two‐spot (big/small) tracking method was used to track the rat's position. The average running speed of the rat during navigation was measured in cm/sec.

### 4.8 Histological analyses

Coronal brain sections were made in the coronal plane with a freezing microtome (50 μm sections in four series). One series was directly mounted on gelatin‐subbed slides, while the other was stored in a cryoprotective solution. The mounted sections were dried overnight, followed by rehydration in a series of ethanol solutions of decreasing concentrations (2 × 100%, 90%, 70%). The sections were then stained with cresyl violet after 2 min in deionized water. Following cresyl violet staining, sections were placed in deionized water and dehydrated in a series of ethanol solutions (70%, 90%, 2 × 100% series). Finally, mounted sections were defatted in xylene and coverslipped with DPX (ThermoFisher, Waltham, MA). The sections were imaged with a Leica DM5000B microscope with a Leica DFC310FX digital camera and Leica Application Suite image acquisition software. Tetrode and bipolar electrode tracks were identified in the acquired images.

### 4.9 Drugs

Rats were subcutaneously injected with NMDA receptor co‐agonist D‐SER (s.c. D‐SER 10 mg/kg, Sigma‐Aldrich, Ireland) in vehicle. D‐SER was mixed in 0.9% of normal saline (NaCl) to achieve 10 mg/ml solution. Control rats received no injections. For LFP recordings, rats were injected with normal saline (NaCl, 0.9% w/x Sodium Chloride BP, Braun, Dublin) as a control for the following day when D‐SER was administered.

### 4.10 Experimental design and statistical analysis

The experimental designs are illustrated in Figures 1a, 2a, 5a, and 6a. The present study is divided into four main sections. First, we investigated place cell activity when rats navigated in a square arena with manipulation of visual stimuli. In this case, the experimental protocol comprised three sessions of 10 min each where rats (*n* rats = 10, *n* place cells = 19) moved from light (L1) to dark (D) and back to light (L2). Second, the protocol was repeated the following day (Day 2) at the same time as the previous day (Day 1). Day 1 was considered as the control for Day 2. In Day 2, a subgroup of rats (*n* rats = 6, n place cells = 9) received (subcutaneously) 10 mg/kg of NMDA receptor co‐agonist D‐SER 30 min before the recording session (see Section 4.9 for further details). Third, an additional group of rats (*n* = 3) were used to test the total power in the LFP and compared with saline. In this case, rats were subcutaneously injected with saline (Day 1) and D‐SER (Day 2). Following the injection, rats were placed into a box where 30 min of LFP recording was performed (see Section 4.4 for more details). For the larger part of the study, when the normal distribution was present, we ran parametric tests (*t* tests, paired), whereas nonparametric tests (Wilcoxon signed‐rank test for paired data) were used otherwise. The Wilcoxon signed‐rank test for paired data was used to compare theta power over the 2 days of recordings before and after the drug. The software GraphPad Prism 8 was used for statistical analysis (*t* test and Wilcoxon signed‐rank test) and graphs. NeuroChaT (Islam et al., 2019) was used to analyse spike properties and relative theta power (see Sections 4.7.1 and 4.7.2, respectively). The analysis for the total power in the 30 min LFP recording was written in Python (v3.7), extending NeuroChaT. The analysis for the total power, the relative delta, theta, and gamma powers between visual cortex and claustrum was written in Python (v3.7).

## CONFLICT OF INTEREST

The authors declare no competing financial interests.

## AUTHOR CONTRIBUTIONS

SOM and ER conceptualized and designed the experiments. ER drafted the initial manuscript, and created figures and tables. SOM, SKM and ER wrote, reviewed, and edited the manuscript. ER and SKM analysed data. SKM coded and developed analysis software. CC performed biochemical analyses. JR performed drug administration. MLM performed histology. SOM supervised and acquired funding.

### PEER REVIEW

The peer review history for this article is available at https://publons.com/publon/10.1111/ejn.15726.

## Data Availability

Single‐unit, LFP, and positional information recorded in this study are freely available on OSF at https://doi.org/10.17605/OSF.IO/3S4MH.
